# Epidemiological Characteristics and Transmissibility for SARS-CoV-2 of Population Level and Cluster Level in a Chinese City

**DOI:** 10.3389/fpubh.2021.799536

**Published:** 2022-01-18

**Authors:** Shanshan Yu, Shufeng Cui, Jia Rui, Zeyu Zhao, Bin Deng, Chan Liu, Kangguo Li, Yao Wang, Zimei Yang, Qun Li, Tianmu Chen, Shan Wang

**Affiliations:** ^1^State Key Laboratory of Molecular Vaccinology and Molecular Diagnostics, School of Public Health, Xiamen University, Xiamen, China; ^2^Chaoyang District Center for Disease Prevention and Control, Beijing, China; ^3^Public Health Emergency Center, Chinese Center for Disease Control and Prevention, Beijing, China

**Keywords:** COVID-19, transmissibility, population-level, cluster-level, mathematical model

## Abstract

**Background:**

To date, there is a lack of sufficient evidence on the type of clusters in which severe acute respiratory syndrome coronavirus 2 (SARS-CoV-2) is most likely to spread. Notably, the differences between cluster-level and population-level outbreaks in epidemiological characteristics and transmissibility remain unclear. Identifying the characteristics of these two levels, including epidemiology and transmission dynamics, allows us to develop better surveillance and control strategies following the current removal of suppression measures in China.

**Methods:**

We described the epidemiological characteristics of SARS-CoV-2 and calculated its transmissibility by taking a Chinese city as an example. We used descriptive analysis to characterize epidemiological features for coronavirus disease 2019 (COVID-19) incidence database from 1 Jan 2020 to 2 March 2020 in Chaoyang District, Beijing City, China. The susceptible-exposed-infected-asymptomatic-recovered (SEIAR) model was fitted with the dataset, and the effective reproduction number (*R*_*eff*_) was calculated as the transmissibility of a single population. Also, the basic reproduction number (*R*_0_) was calculated by definition for three clusters, such as household, factory and community, as the transmissibility of subgroups.

**Results:**

The epidemic curve in Chaoyang District was divided into three stages. We included nine clusters (subgroups), which comprised of seven household-level and one factory-level and one community-level cluster, with sizes ranging from 2 to 17 cases. For the nine clusters, the median incubation period was 17.0 days [Interquartile range (IQR): 8.4–24.0 days (d)], and the average interval between date of onset (report date) and diagnosis date was 1.9 d (IQR: 1.7 to 6.4 d). At the population level, the transmissibility of the virus was high in the early stage of the epidemic (*R*_*eff*_ = 4.81). The transmissibility was higher in factory-level clusters (*R*_0_ = 16) than in community-level clusters (*R*_0_ = 3), and household-level clusters (*R*_0_ = 1).

**Conclusions:**

In Chaoyang District, the epidemiological features of SARS-CoV-2 showed multi-stage pattern. Many clusters were reported to occur indoors, mostly from households and factories, and few from the community. The risk of transmission varies by setting, with indoor settings being more severe than outdoor settings. Reported household clusters were the predominant type, but the population size of the different types of clusters limited transmission. The transmissibility of SARS-CoV-2 was different between a single population and its subgroups, with cluster-level transmissibility higher than population-level transmissibility.

## Introduction

The global pandemic of the coronavirus disease 2019 (COVID-19) has had a serious impact on public health systems. COVID-19 differs significantly from infectious diseases such as SARS and influenza in terms of epidemiological characteristics (1). As for the transmissibility, COVID-19 is more infectious than influenza, but weaker than measles (2).

Of those people who presented with symptoms significant enough to be classed as patients, the majority (81%) presented with mild to moderate symptoms, while 14% presented with severe symptoms, and 5% suffer critical symptoms (3). Older people are at a higher risk of developing severe symptoms (4). It is necessary to estimate the transmissibility of COVID-19 to determine the severity and size of the pandemic, and to design appropriate interventions and responses to protect the population and control the spread of the disease (5). A systematic review found that the population-level transmissibility of SARS-CoV-2 was 3.32 (95% CI, 2.81 to 3.82) (6), with WHO estimates ranging from 1.4 to 2.5 (7). The transmissibility of the Delta variant, is substantially higher. Among five studies cataloged in October 2021, the mean estimated basic reproduction number (*R*_0_) was 5.08 for Delta (8).

Most studies for cluster-level epidemic focused on the epidemiological characteristics of transmission chains and case profiles across generations (9–11). Researchers have found many examples of SARS-CoV-2 clusters associated with indoor settings, with many reports from households, few from schools, and increasing reports from hospitals and elderly care settings across Europe (12). Limited studies have focused on the transmissibility of the virus in cluster-level outbreaks. A SEIR (susceptible-exposed-infectious-removed) based modeling study calculated an *R*_0_ of 3.06 (95% CI: 2.64–3.51) for 15 clusters (13). Another cluster-based study found a mean transmission rate of 1.86 per case among family members (14).

To amplify the reasons for the reported heterogeneity in transmission: the number of people infected by one infected person generally varies (15); as only 10 to 20% of the population are responsible for the disease's spread (16). It often spreads in clusters, where infections can be traced back to an index case or geographical location (17). In these instances, superspreading events often occur, where many people are infected by one person (15). Thus, we need to know in which types of clusters the virus is most likely to be transmitted. Notably, the differences in the epidemiological characteristics and transmissibility of cluster-level and population-level outbreaks remain unclear. Following the current removal of suppression measures in China, identifying the characteristics of both levels including epidemiology and transmission dynamics allows us to develop better surveillance and control strategies.

In this study, we first compared and analyzed the differences in epidemiological characteristics of COVID-19, including the population distribution, incubation period, and time interval. We then proposed the SEIAR (Susceptible-Exposed-Infectious-Asymptomatic-Removed) model (18–20) for calculation of population-level transmissibility (PLT). After a rigorous investigation to clarify the relationship between primary and secondary cases, we calculated the cluster-level transmissibility (CLT) by definition (21). CLT is defined as the expected number of cases infected by a single case during the entire infectious period.

## Methods

### Study Design

**Figure 1:**
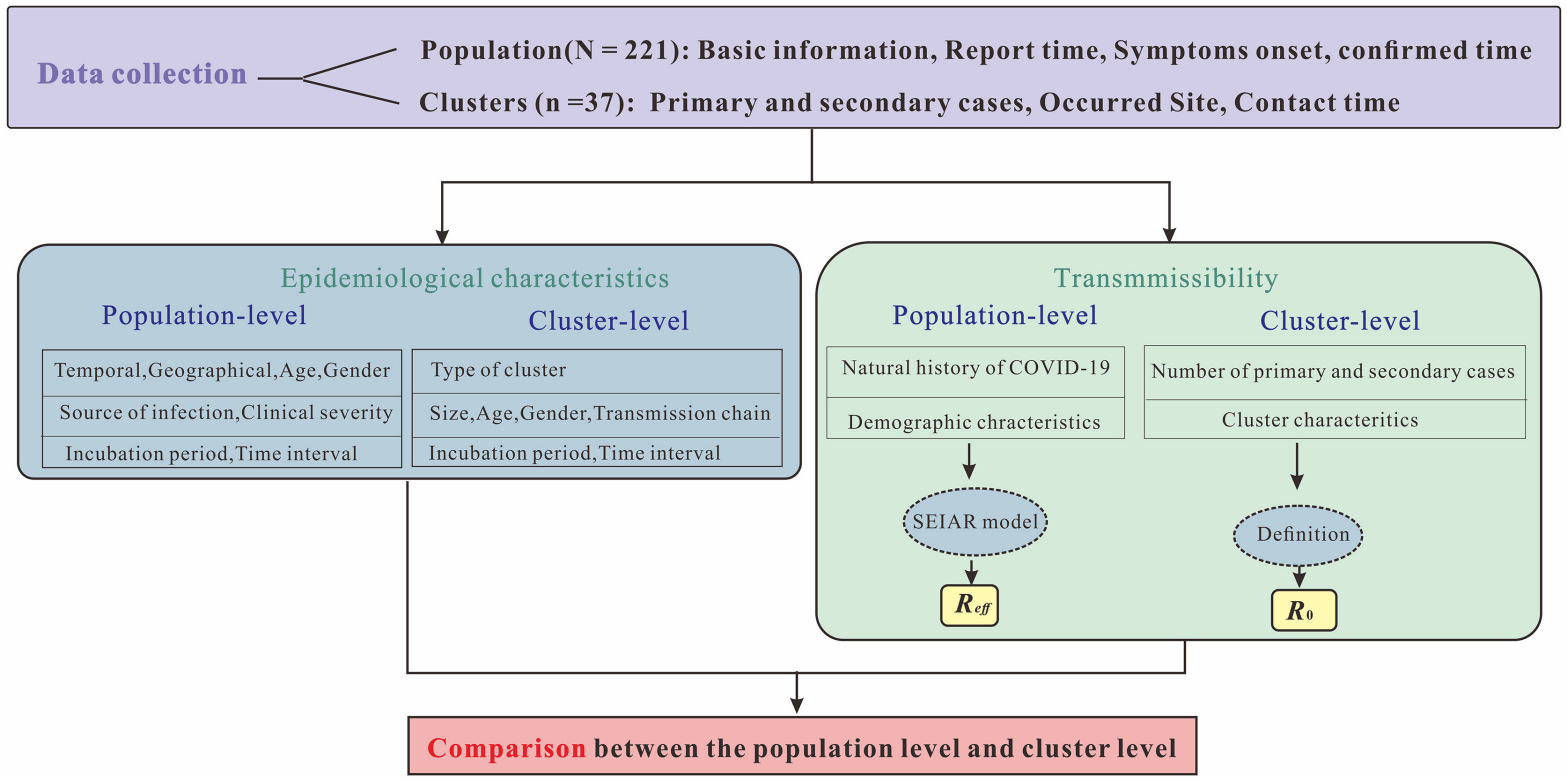
Research technical route. The letter N represents number of symptomatic and asymptomatic infected cases in Chaoyang District, China in 2020, while n represents number of clusters reported. Symptoms onset indicates date of illness onset for symptomatic cases, while report date indicates first RT-PCR positive result for asymptomatic infections. Confirmed time indicates date of diagnosis for both infections.

This study was divided into four sections (Figure 1). The first section briefly described the epidemiological characteristics of reported COVID-19 cases, including temporal, geographical, age and gender distributions, source of infection, clinical severity as well as incubation period and time interval. In the second section, the SEIAR model was established for calculating *R*_*eff*_ as PLT (i.e., population-level transmissibility). The calculation of CLT (i.e., cluster-level transmissibility) by definition of *R*_0_ were presented in the third section. The final section compared cluster-level and population-level transmissibility.

### Data Sources

**Figure 2:**
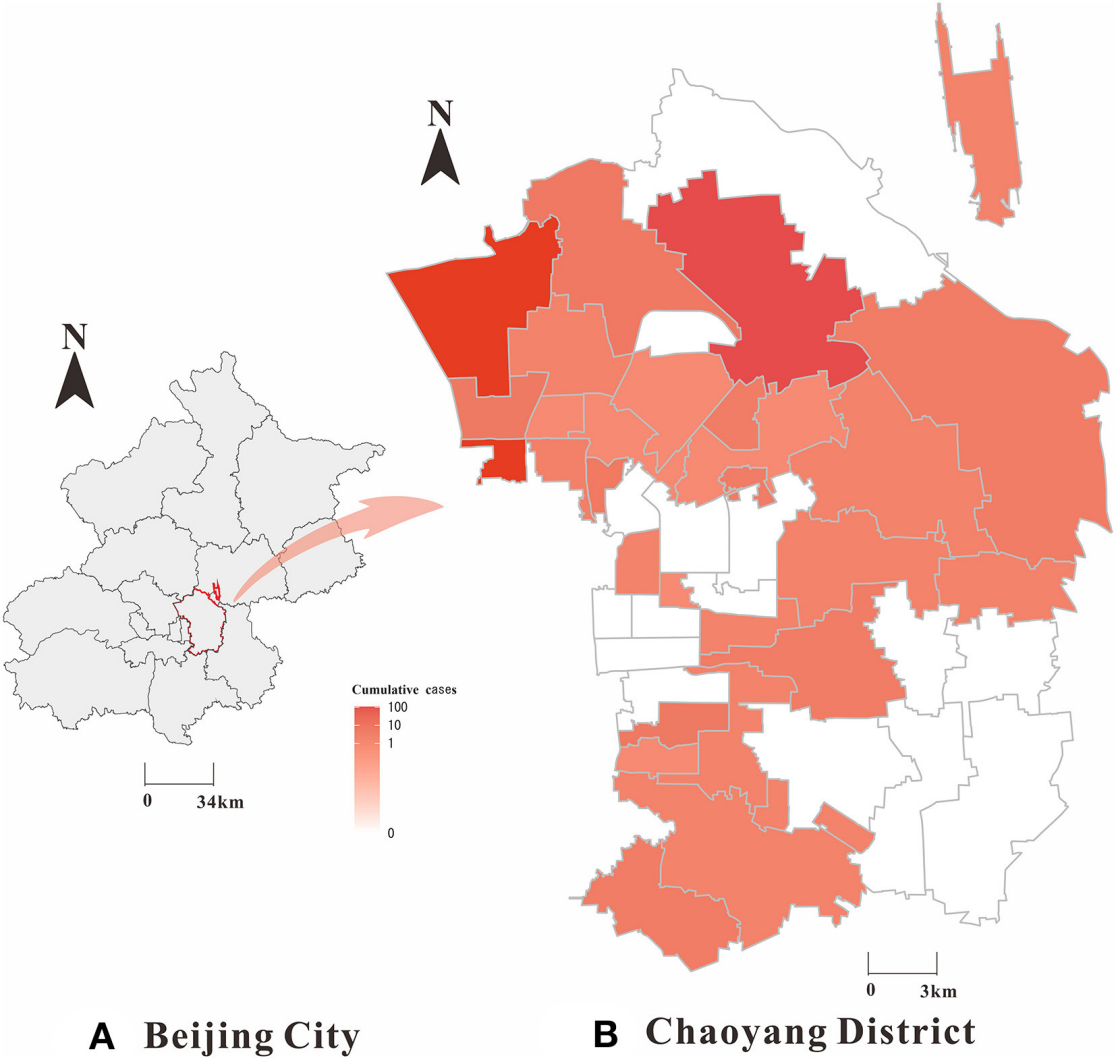
Geographical distribution of SARS-CoV-2 symptomatic and asymptomatic infected cases in Chaoyang District, China (2020). **(A)** Beijing City; **(B)** Chaoyang District; The map depicted in this figure was taken from Wikimedia Commons (http://commons.wikimedia.org/wiki/Main_Page).

In this study, we collected daily reported COVID-19 cases in Chaoyang District (Figure 2) from 1 January 1 2020 to 31 December 2020 from the Chinese Center for Disease Control and Prevention through the National Notifiable Disease Surveillance System (22). The variables included in the COVID-19 dataset mainly comprised gender, age, occupation, the site of residence, symptomatic infections, asymptomatic infections, date of onset, date of diagnosis, date of discharge or death, and severity of disease. Demographic data were obtained from the Chinese Statistical Yearbook. For definitions of symptomatic and asymptomatic infected cases and clinical types, we refer to the Prevention and Control Program for COVID-19 (23) published by the National Health Council.

### Definitions Used for Each of Our Transmission Setting Types

In our study, types of clusters were defined according to where they occurred (Table 1). There are basically three types of places where a person stays: one leaves his or her home and go to work, and all the places he or she passes through are called communities. Clusters at the household level imply that these outbreaks occurred at home. Factory-level clusters refer to places like offices or schools, which consist of various places where people work. Community-level clusters mean that these outbreaks occur anywhere outside the home and workplace, such as supermarkets, buses, etc.

**Table 1:** Definitions used for each of our transmission setting types.

**Transmission setting**	**Definition**
Household	Transmission between individuals in a shared living space
Factory	In the workplace, typically an office.
Community	Where transmission occurs on public property and does not fall into any of the above two settings e.g., supermarkets, buses, hotel, park, etc.

### Estimation of PLT Based on SEIAR Model

**Figure 3:**
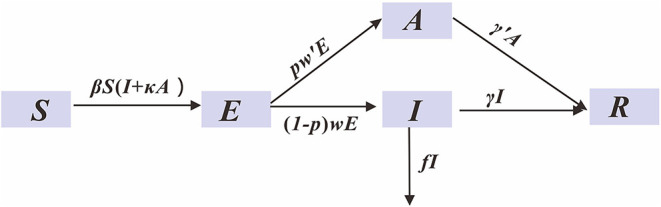
SEIAR model for simulating COVID-19.

We take the actual data from 1 January 1 2020 to 2 March 2020 to fit the SEIAR model. Our model (Figure 3) was built based on the approach described in our previous paper (18) (See Supplementary Materials for detailed information of this model). The estimation of the parameters and the initial values of the variables were shown in Table 2. We calculated the PLT using this SEIAR model.

**Table 2:** The definition and values of parameters in SEIAR model of COVID-19 in Chaoyang District, China (2020).

**Parameter**	**Definition**	**Value**	**Range**	**Source**
β	Transmission relative rate	-	≥ 0	Curve fitting
κ	Relative transmissibility rate of asymptomatic to symptomatic individuals	0.5000	0–1	(24)
*p*	Proportion of the asymptomatic	0.0357	0–1	Actual data
*1/ω*	Incubation of symptomatic	7	0–1	(23)
*1/ω'*	Incubation of asymptomatic	5	0–1	(23)
*1/γ*	Infectious period of symptomatic	25	0–1	Actual data
*1/γ'*	Infectious period of asymptomatic	6	0–1	(24)
*f*	Fatality of the disease	0.0000	0–1	Actual data

***-**, Represents the result to be fitted*.

At population-level epidemics, the population was not completely susceptible or the intervention measures were taken, the effective reproductive number (*R*_*eff*_ or *R*_*t*_) was used to express the transmissibility of infectious diseases. *R*_*eff*_ is defined as the expected number of secondary cases arising from a single infected individual at time t, with a given level of immunity in the population. In this study, *R*_*eff*_ is calculated by the second-generation matrix method:


(1) $$\begin{array}{r} \underset{dr\to \infty }{\lim}R_{eff}=\beta S\left(\frac{1-p}{\gamma +f}+\frac{\kappa p}{\gamma }\right) \end{array}$$

where β denotes the infection rate coefficient, which is the average number of contacts per person per time multiplied by the probability of disease transmission in a contact between susceptible and an infectious subjects; *S* is the susceptible population; *p* denotes the proportion of the asymptomatic infections; γ denotes the probability of an infectious individual recovering within the average infectious time period, and 1/γ means the infectious period of symptomatic infections; *f* means the fatality of the disease; κ indicates the relative transmission rate of asymptomatic infections to symptomatic infections.

### Estimation of CLT Based on Definition

At cluster-level epidemics, the basic reproduction number (*R*_0_) was suitable for evaluating the transmissibility quantitatively. Based on the nine clusters according to the inclusion and exclusion criteria (See Supplementary Figure S1 for detailed information), we aimed to calculate the CLT based on the definition of *R*_0_ (See Supplementary Materials for definition of *R*_0_). This calculation method required a detailed investigation to clarify the relationship between the cases which was available in studies of clusters. We directly divided the number of cases in the (*n* + 1) generation by the number of cases in the (n) generation. For example, Ebola has an *R*_0_ of two, so on average each person with Ebola passes it on to the two other people (See Supplementary Figure S2). The formula is as follows,


(2) $$R_{0}=\frac{N^{(n+1)th}}{N^{nth}}$$

where *N* denotes the number of cases, *N*^(n+1)*th*^ means the number of cases in the (*n*+1) generation, *N*^nth^ means the number of cases in the (n) generation. Cases mean symptomatic or asymptomatic infections.

### Statistical Analysis

Microsoft Excel 2019 software (Microsoft Corp, USA) was used for the entry and management of the relevant data. We performed all statistical analysis with IBM SPSS Statistics for Windows, version 26.0 (IBM Corp., Armonk, N.Y., USA), and *p* < 0.05 (typically ≤ 0.05) was statistically significant. R 3.6.3 software and Data Map 6.2 software (Microsoft Corp, USA) were used for spatial map analysis. The software used in the model simulations was Berkeley Madonna 8.3.18 (developed by Robert Macey and George Oster at the University of California, Berkeley. Copyright©1993-2001 Robert I. Macey and George F. Oster). The differential equations were solved by the fourth-order Runge-Kutta method, and model convergence was based on the least root mean square (LRMS). The coefficient of determination (*R*^2^) was used to assess the goodness of fit (24, 25).

## Results

### Population-Level Epidemiological Characteristics

**Figure 4:**
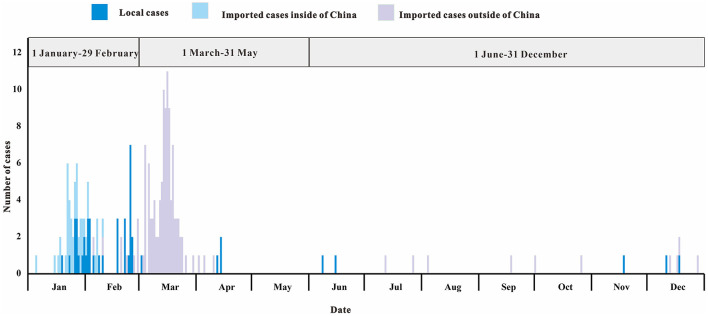
Daily number of SARS-CoV-2 infections stratified by source of infection in Chaoyang District, China (2020).

In 2020, the COVID-19 outbreak in Chaoyang District was divided into three different stages of prevention and control (Figure 4). The first stage (January 1 2020 to February 29 2020) was the control of cases from Chinese cities including Wuhan City. The second stage (March 1 2020 to May 31 2020) was to control cases from abroad. The third stage (June 1 2020 to December 31 2020) was to control local clusters caused by imported cases.

We found a balanced gender distribution (sex ratio = 104:107) among COVID-19 cases (Table 3). The majority of patients were between 20 and 59 years of age, with a median age of 33 years. Critical and severe cases (21/211, 10%) were rare. The rate of asymptomatic infection was 0.004 per 1,000 people.

**Table 3:** Characteristics of cases^a^ in three stages in Chaoyang District, China (2020).

**Characteristics**		**1 January-29 February**	**1 March-31 May**	**1 June-31 December**	**In total**
		***n* = 87**	***n* = 109**	***n* = 15**	***n* = 211**
Source of infection					
	Local cases	38(43.7)	4(3.7)	5(33.3)	47(22.3)
	Imported cases inside of China	40(46.0)	0(0)	0(0)	40(19.0)
	Imported cases outside of China	9(10.3)	105(96.3)	10(66.7)	124(58.7)
Sex					
	Male	49(56.3)	46(42.2)	9(60.0)	104(49.3)
	Female	38(43.7)	63(57.8)	6(40.0)	107(50.7)
Age, years					
	Median(IQR)	42 (31–54)	24 (20–39)	36 (23–48)	33 (22–47)
	0–19	7 (8)	20(18.3)	2(13.3)	29(13.7)
	20–39	29(33.3)	62(56.9)	7(46.7)	98(46.4)
	40–59	38(43.7)	24 (22)	4(26.7)	66(31.3)
	60–79	11(12.6)	3(2.8)	2(13.3)	16(7.6)
	≥80	2(2.3)	0(0)	0(0)	2(1.0)
Clinical severity					
	Symptomatic patients
	Mild patients	36(41.4)	56(51.4)	8(53.3)	100(47.4)
	Moderate patients	29(33.3)	47(43.1)	6 (40)	82(38.9)
	Severe patients	7(8.0)	0(0)	0(0)	7(3.3)
	Critical patients	8(9.2)	6(5.5)	0(0)	14(6.6)
	Asymptomatic patients
		7(8.0)	0(0)	1(6.7)	8(3.8)

*Data are presented as no. (%) of cases unless otherwise indicated*.

a *Cases are comprised of symptomatic and asymptomatic infected cases*.

*b: IQR is the abbreviation of Interquartile range*.

For the epidemiological parameters (Table 4), we set a fixed incubation period of 5.0 to 7.0 days [d] for symptomatic and asymptomatic cases of infection, respectively, based on previous study (23). The shortest time interval was 0.1 d, the longest was 33.2 d, and the median interval was 3.4 d [interquartile range (IQR), 1.6–7.0 d]. The time interval is longer for symptomatic infections [Median (M), 3.4 d] than for asymptomatic infections (M, 0.9 d).

**Table 4:** Population-level epidemiological parameters.

		**Symptomatic patients**	**Asymptomatic patients**	**In total**
Incubation period, days	(2)^a^	5.0	7.0	-
Time interval^b^, days	Min-Max	0.3–33.2	0.1–1.4	0.1–33.2
	Median, IQR	3.4(1.7–7.1)	0.9(0.7–1.0)	3.4(1.6–7.0)

a *The population-level incubation period is fixed based on previous studies*.

b *The dataset we built includes the information on diagnosis date and date of onset as well as report date. For symptomatic infections, time interval is equals to diagnosis date minus date of onset. While for asymptomatic infections, time interval is equals to diagnosis date minus report date*.

### Cluster-Level Epidemiological Characteristics

**Figure 5:**
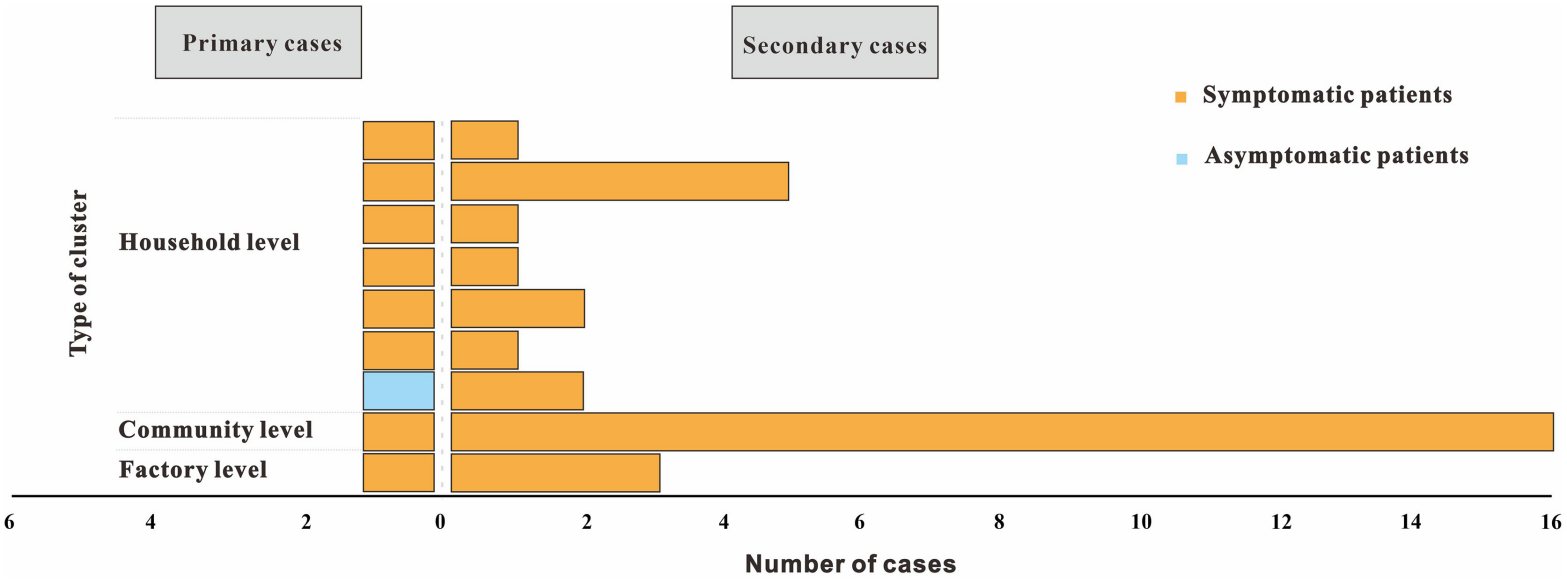
Distributions for type of cluster and presence of symptoms of nine COVID-19 clusters reported from Chaoyang District, China (2020). Type of cluster depends on the setting where exposure took place, with primary cases considered as first generation. Presence of symptoms include symptomatic and asymptomatic infections.

Four clusters (44.4%) reported only one secondary case and another five clusters (55.6%) reported more than two secondary cases. The size of each cluster ranged from 2 to 17 cases (*M* = 2). In 2020, clusters occurred from the initial household to the factory to the community (Figure 5).

For nine clusters, the difference between the shortest (3.0 d) and longest (30.0 d) incubation period was 10 times. The median incubation period is 17.0 d (IQR, 8.4–24.0 d). The shortest time interval was 0 d, while the longest was 32.0 d, and the average time interval was 1.8 d (IQR, 1.7–6.4 d). The factory-level cluster had the longest incubation period (M, 24.0 d). The shortest time interval was 0 d (IQR, 3.2–9.6d) for the household-level clusters (Table 5).

**Table 5:** Cluster-level epidemiological parameters.

**Type of cluster**		**Household**	**Factory**	**Community**	**In total**
Incubation period, days	Min-Max	3.0–17.0	11.0–30.0	4.0–6.0	3.0–30.0
	Median, IQR	9.5(4.5–15.0)	24.0(23.0–24.0)	5.0(4.5–5.5)	17.0(8.4–24.0)
Time interval, days	Min-Max	0.0–32.0	1.0–6.2	0.6–2.0	0.0–32.0
	Median, IQR	0.0(3.2–9.6)	2.0(1.5–2.1)	1.9(1.6–2.0)	1.8(1.7–6.4)

### Population-Level Transmissibility for SARS-CoV-2

**Figure 6:**
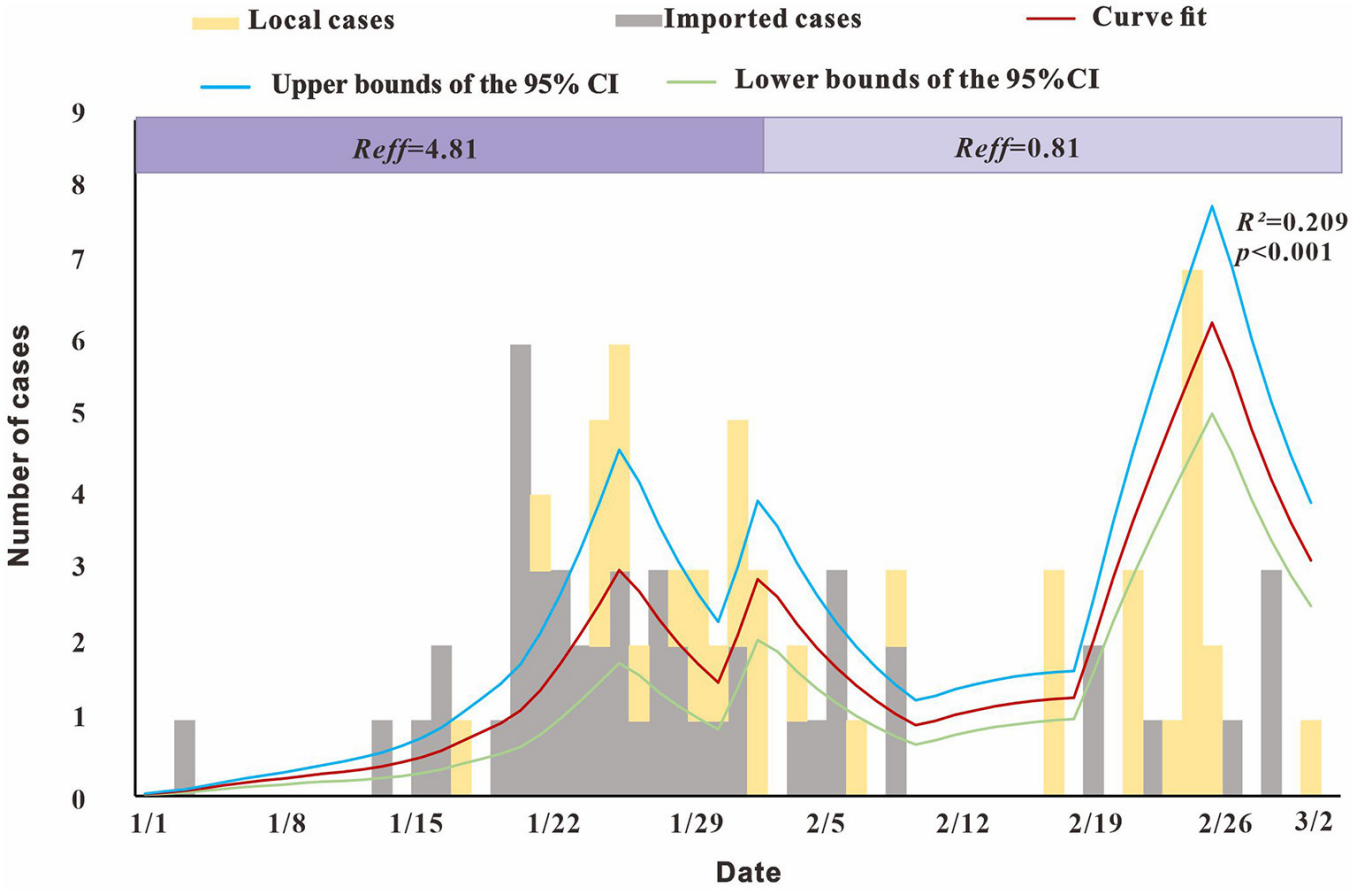
Fitting results of the SEIAR model and the data of the actual secondary cases of SARS-CoV-2 infections.

The modeling results showed (Figure 6) that *R*_*eff*_ reached 4.81 before February 1, that is, an infected person can infect an average of 4.81 susceptible persons during the average incubation period. After February 1, *R*_*eff*_decreased to 0.81, with a reduction of almost 100%, indicating that the outbreak was gradually controlled.

### Cluster-Level Transmissibility for SARS-CoV-2

**Figure 7:**
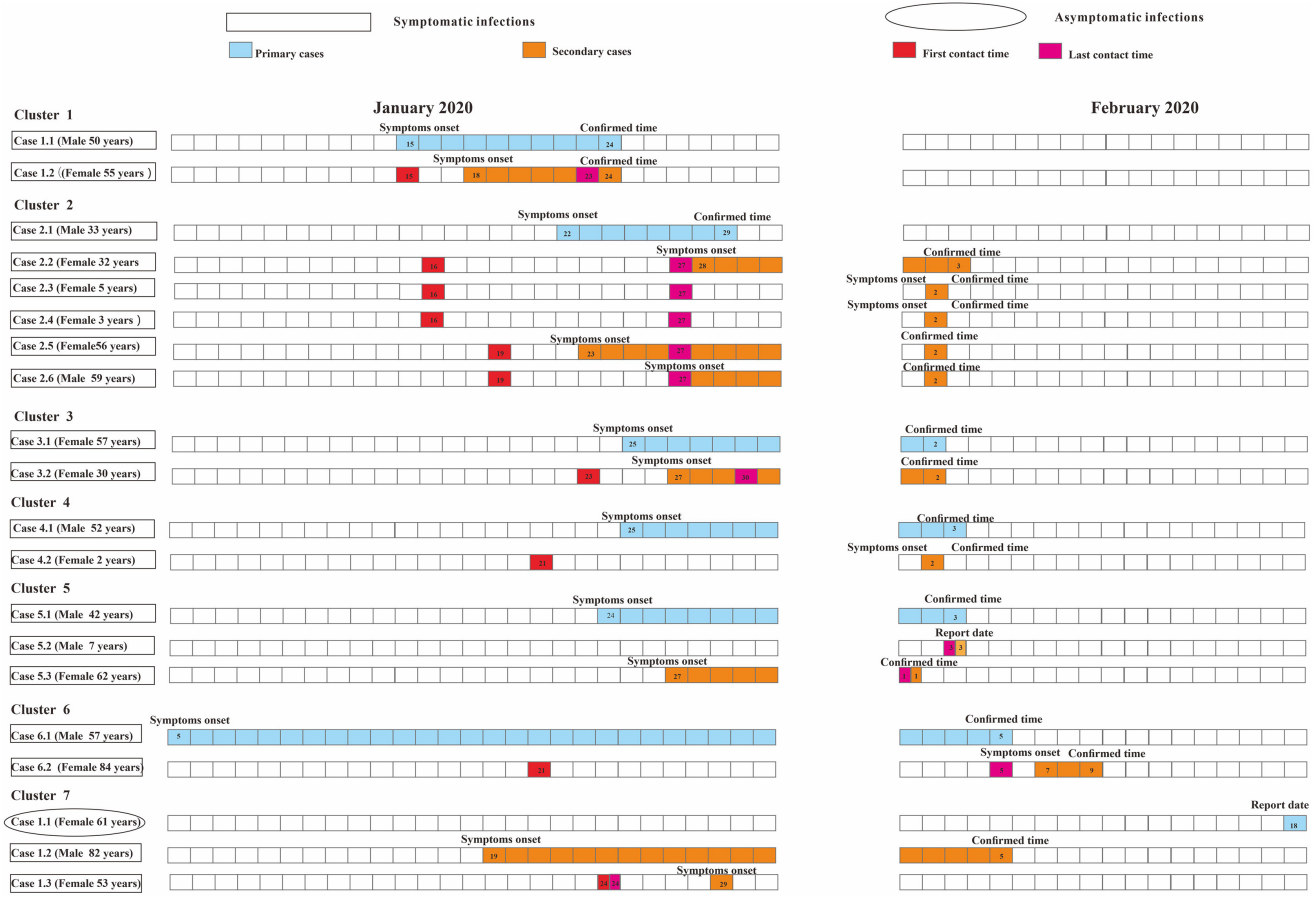
Timing of transmission events in seven household-level clusters. Square symbols indicate symptomatic infections and circular symbols indicate asymptomatic infections. Blue grids indicate primary case and purple grids indicate secondary cases. Age, sex, and generation in a cluster are shown for each SARS-CoV-2 infected individuals, with information of date of illness onset (symptoms onset) for symptomatic cases and date of the first RT-PCR positive result (report date) for asymptomatic infections and date of diagnosis (confirmed time) for both infections. Timeline of transmission also includes the first and last contact time between the primary case and secondary cases. Except for community-level cluster where there is no contact time due to contacts *via* public objects, all other types of clusters have first contact time and last contact time between secondary cases and primary cases. The illustrations of Figure 7 and Figure 8 are the same as here.

**Figure 8:**
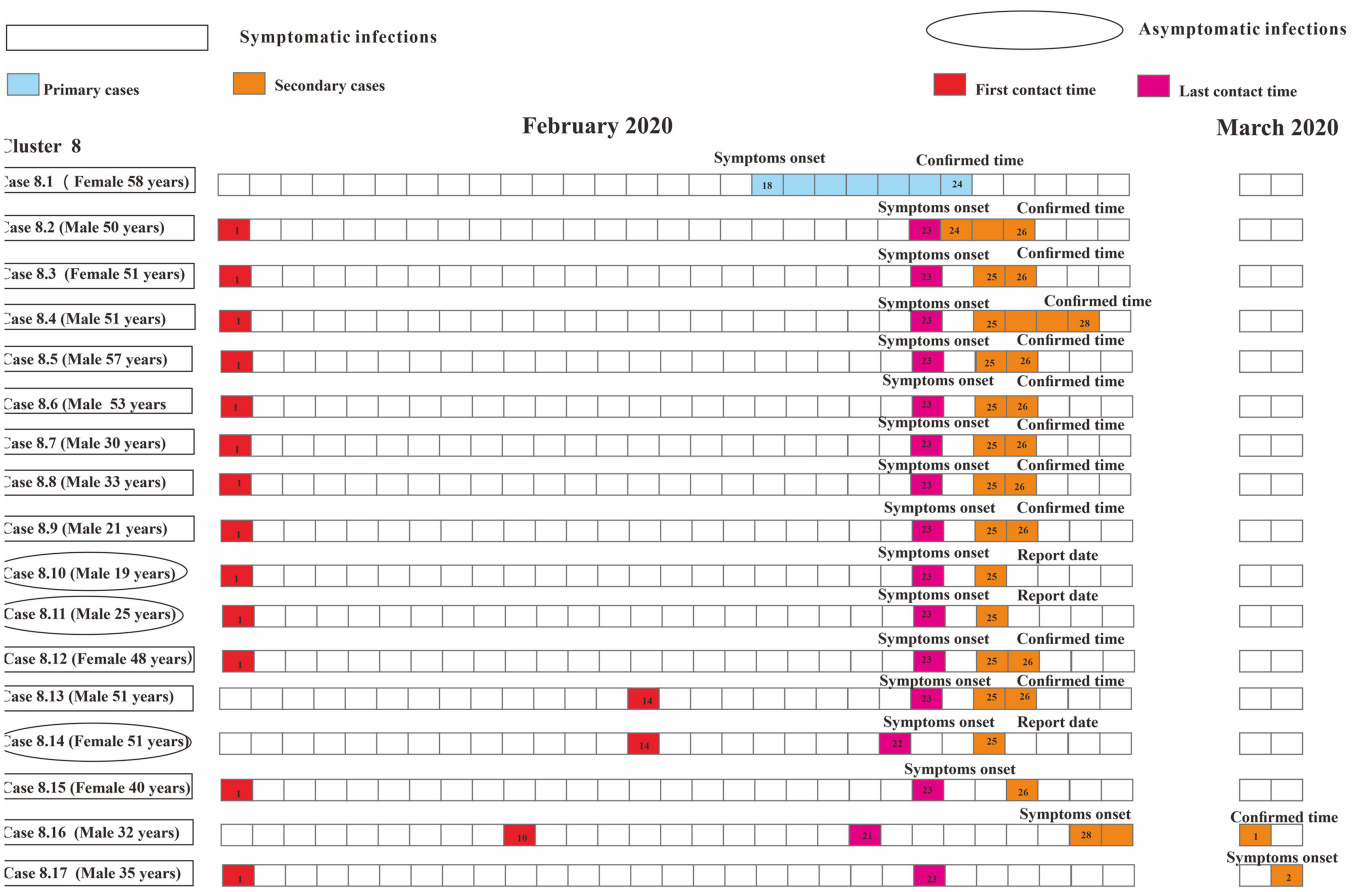
Timing of transmission event in one factory-level cluster.

**Figure 9:**
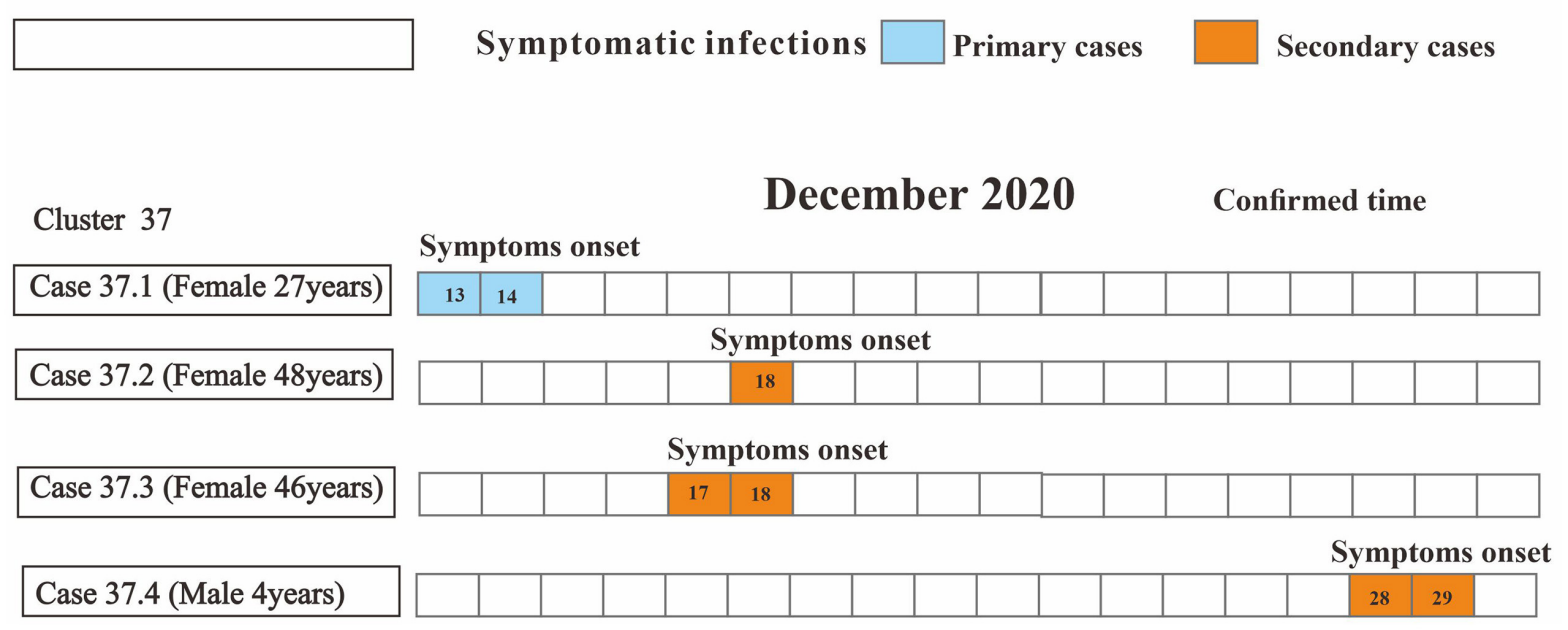
Timing of transmission event in one community-level cluster.

As for the household-level cluster, the median *R*_0_ was one (Figure 7). Sixteen individuals were diagnosed with COVID-19 in this factory-level cluster, with *R*_0_ of 16(Figure 8). Three individuals in this community-level cluster were diagnosed with COVID-19, with *R*_0_ of three (Figure 9). The factory-level cluster (*R*_0_ = 16) had higher transmissibility than the community-level cluster (*R*_0_ = 3) and the household-level cluster (*R*_0_ = 1).

## Discussion

### Analysis of Population-Level Epidemiological Characteristics

We found that the population-level epidemic was characterized by three stages with different primary prevention and control populations, which was similar to previous studies (26). In our study, we set 5–7 d (23) as the population-level incubation period, which was consistent with previous work. The results of Lauer et al. (27) showed a mean incubation period of 5.2 d (95% CI: 4.4–6.0 d), a Japanese study showed a mean incubation period of 5 d (95% CI: 2–14 d) (28), a Dutch study estimated mean incubation period of 6.4 d (95% CI: 5.6–7.7 d) (29), and one study evaluated a median incubation period of 7 d (30).

### Analysis of Cluster-Level Epidemiologic Characteristics

The setting where clusters occurred also changed, from household to factory and to community. In the early stage, cold weather and isolation measures contributed to the survival of the virus and the increased risk of intra-household transmission (31, 32). The long chain of transmission at factory-level cluster could be the failure to effectively disinfect and maintain social distances once people started returning to the workplace. Outbreaks occur in different workplaces when it is difficult to maintain the recommended distance of at least 2 meters (33, 34). Shared facilities (e.g., canteen and dressing rooms), transportation and accommodations may also lead to transmission (35). Coronavirus infections are characterized by intermittent shedding, long incubation periods, and sampling locations can affect detection results, therefore there were cases undetected (36), causing a community-level transmission after removal of isolation at the end of 2020.

For household clusters, the length of the incubation period was similar to previous studies (4, 37). The longer incubation period for this factory-level cluster may be related to the increase in asymptomatic infections, individual heterogeneity, and virus mutation. This community-level cluster occurred at a late stage. At this time, the intensity and efficiency of nucleic acid detection has improved considerably compared to the previous stage, so that the time to detect cases is shorter than that in the earlier household-level clusters. In addition, the discrepancy may be due to lack of accuracy in the investigation of the time of contact with the infectious agent and lack of large sample size.

### Analysis of Population-Level Transmissibility of SARS-CoV-2

Our *R*_*eff*_ value was higher than those estimated by the SAPHIRE (Susceptible–unascertained-presymptomatic-hospitalized-infectiousness–infectious–recovered-exposed) model for early outbreaks in Wuhan City (38), and by World Health Organization (*R*_*eff*_ = 1.4–2.5) and by other studies for Beijing City (*R*_*eff*_ <3) (7, 39, 40). While our estimation was similar to the values estimated for the SARS (severe acute respiratory syndrome) epidemic in Beijing, China (*R*_0_ = 4.91) (41), and for MERS (Middle East respiratory syndrome) in Jeddah, Saudi Arabia (*R*_0_ = 3.5 to 6.7) (42).

There are several possible reasons for this. First, this high *R*_*eff*_ was calculated for the early stages, where preventive measures were inadequate due to the small sample size of cases and the lack of awareness about the disease. Additionally, the estimates depend on social and demographic variables, the estimation method used, the validity of the underlying assumptions, and the biology of the infectious agent. Third, modeling methods also contribute to the difference (43). Finally, estimates of *R*_*eff*_ may be wrong due to insufficient data, thus precisely estimating *R*_*eff*_ is rather difficult.

### Transmission Risks in Different Clusters

The setting with the highset number of reported clusters of SARS-CoV-2 transmission was households, but household-level transmissibility (*R*_0_ = 1) was not the highest. The basic reproduction number may be limited by the number of people in each cluster. For example, the number of family members determines the upper limit of secondary cases in household transmission. Several outbreak investigation reports suggest that SARS-CoV-2 transmission can be particularly effective in crowded, confined indoor spaces (12).

Patients involved at the factory-level were more effective in contacting susceptible populations than at the household-level, leading to high transmissibility. Multiple outbreaks of COVID-19 have been observed in several occupational settings, including slaughterhouses, meat processing plants, mines and building sites (12, 44). Possible factors contributing to clusters in occupational settings are listed below. Studies have shown that in Europe, more than 80% of working time is spent indoors, and changes in socioeconomic and demographic conditions have led to different work-day patterns indoors (45). Participating in meetings and sharing the same office space has been reported in literature as a risk factor for contracting COVID-19 (33, 46).

### Comparison of Epidemiological Characteristics and Transmissibility Between Population-Level and Cluster-Level

Both population-level and cluster-level epidemic were characterized by multiple stages. The incubation period and time interval were similar to previous studies. Population-level transmissibility (*R*_*eff*_ = 4.81) was intermediate to cluster level (*R*_0_ = 1~16). Frequent close contacts more or less create opportunities for increased transmission risk, such as indirect contact transmission and respiratory droplet transmission. *R*_0_ values differed in different subgroups of a single population. In fact, the total value of *R*_*eff*_ in a population is the average of the *R*_0_ subtypes in that population (47). We need to note that even if the total value of *R*_0_ in a population is moderate (*R*_*eff*_ = 4.81), the transmission potential of some subgroups in that population may still be high, for instance, the factory-level *R*_0_ is equal to 16. Besides, the basic reproduction number is affected by several factors, including the duration of infection of the affected person, the infectiousness of the microorganism, and the number of susceptible individuals in the population with which the infected people is in contact (48).

In the early stage of the disease outbreak, *R*_*eff*_ was estimated based on a differential equation model of the overall epidemic data. However, it only represents the average epidemic pattern, and it ignores the heterogeneity of infectivity within populations due to structural differences in different settings such as households, factories and communities. Based on detailed data obtained from epidemiological investigation, *R*_0_ can be calculated by definition. This indicator excludes the possibility of close contacts contacting other infected persons and therefore has some advantages in analyzing the transmissibility of clusters.

### Implications for Prevention and Control Measures

Following the current lifting of suppression measures in China, the key population is mainly imported cases, and asymptomatic infections. Active screening and expanded nucleic acid testing for the population and high-risk infection subgroups are needed, especially indoor settings. And epidemiological investigation and traceability efforts should be conducted to prevent and reduce the occurrence of indoor and outdoor clusters.

In addition, implementing the joint screening strategy is urgent in the next step of control. Some regions (49) in China have begun to adopt a combined screening strategy of 14-day centralized isolation, 7-day centralized or home isolation and multiple nucleic acid and serum total antibody screening (50, 51) for inbound personnel, which can effectively improve the detection rate and reduce the transmission risk.

### Limitations and Future Directions

This study has limitations. First, we only used a limited number of samples. A larger sample might have led to a higher generalization of our results. Second, our research focused on natural history of disease, and it might be important to include other factors as well. Studies (38, 52–54) have suggested factors that would have an impact on the epidemic situation, such as climate, use of mask, inflow and outflow of population, clinical classification of confirmed cases, reinfection, virus mutation, presymptomatic infection, undiagnosed infections, hospitalization or home isolation measures for confirmed cases.

In subsequent studies, consideration of the above factors can be added to make the dynamic model more consistent with the actual situation. In addition, a household-community-factory-based COVID-19 transmission scenario could be constructed based on our calculated transmissibility of the three scenarios.

## Conclusions

In Chaoyang District, the epidemiological features of SARS-CoV-2 showed multi-stage pattern. Many clusters were reported to occur indoors, mostly from households and factories, and few from the community. The risk of transmission varies by setting, with indoor settings being more severe than outdoor settings. Reported household clusters were the predominant type, but the population size of the different types of clusters limited transmission. The transmissibility of SARS-CoV-2 was different between a single population and its subgroups, with cluster-level transmissibility higher than population-level transmissibility.

## Data Availability Statement

The raw data supporting the conclusions of this article will be made available by the authors without undue reservation.

## Author Contributions

SY, SC, JR, QL, TC, SW, ZZ, BD, CL, KL, YW, and ZY had full access to all of the data in the study and take responsibility for the integrity of the data and the accuracy of the data analysis. SW, TC, and QL were responsible for its conception and design, made critical revision of the manuscript for important intellectual content, and contributed equally to the supervisions of this work. SC and SW collected the data. SY, SC, JR, ZZ, BD, CL, KL, YW, and ZY were responsible for the analysis, or interpretation of data. SY, SC, and JR drafted the manuscript. All authors contributed to the article and approved the submitted version.

## Funding

This study was partly supported by the Bill and Melinda Gates Foundation (Grant Number: INV-005834). The funder had no role in study design, data collection and analysis, decision to publish, or preparation of the manuscript.

## Conflict of Interest

The authors declare that the research was conducted in the absence of any commercial or financial relationships that could be construed as a potential conflict of interest.

## Publisher's Note

All claims expressed in this article are solely those of the authors and do not necessarily represent those of their affiliated organizations, or those of the publisher, the editors and the reviewers. Any product that may be evaluated in this article, or claim that may be made by its manufacturer, is not guaranteed or endorsed by the publisher.

## References

[1] Petersen E Koopmans M Go U Hamer DH Petrosillo N Castelli F. Comparing SARS-CoV-2 with SARS-CoV and influenza pandemics. Lancet Infect Dis. (2020) 20:e238–e44. 10.1016/S1473-3099(20)30484-9 32628905 PMC7333991

[2] How COVID-19 Spreads. Centers for Disease Control and Prevention. Available online at: https://www.cdc.gov/coronavirus/2019-ncov/prevent-getting-sick/how-covid-spreads.html. (accessed July 14, 2021).

[3] Interim Clinical Guidance for Management of Patients with Confirmed Coronavirus Disease (COVID-19). US Centers for Disease Control and Prevention (CDC) (2020). Available online at: https://www.cdc.gov/coronavirus/2019-ncov/hcp/clinical-guidance-management-patients.html (accessed April 19, 2020).

[4] Response EWGfNE. The epidemiological characteristics of an outbreak of 2019 novel coronavirus diseases (COVID-19) in China. Chin J Epidemiol. (2020) 41:145–51. 10.46234/ccdcw2020.03234594836

[5] Kwok KO Tang A Wei VWI Park WH Yeoh EK Riley S. Epidemic models of contact tracing: systematic review of transmission studies of severe acute respiratory syndrome and middle east respiratory syndrome. Comput Struct Biotechnol J. (2019) 17:186–94. 10.1016/j.csbj.2019.01.003 30809323 PMC6376160

[6] Alimohamadi Y Taghdir M Sepandi M. Estimate of the basic reproduction number for COVID-19: a systematic review and meta-analysis. J Prev Med Public Health. (2020) 53:151–7. 10.3961/jpmph.20.076 32498136 PMC7280807

[7] Mahase E. China coronavirus: what do we know so far? BMJ. (2020) 368:m308. 10.1136/bmj.m308 31980434

[8] Liu Y Rocklöv J. The reproductive number of the Delta variant of SARS-CoV-2 is far higher compared to the ancestral SARS-CoV-2 virus. J Travel Med. (2021) 28. 10.1093/jtm/taab124 34369565 PMC8436367

[9] Ye Lixia WH Lu Huaichu Chen Bingbing Zhu Yingying Gu Shaohua Wang Jianmei Pan Xingqiang Fang Ting Dong Hongjun. Investigation of a cluster epidemic of COVID-19 in Ningbo. Chin J Epidemiol. (2020) 41:2029–33. 10.3760/cma.j.cn112338-20200316-00362 32397698

[10] Zhang Jinzhong ZP Han D Wang W Cui C Zhou R Xu K. Investigation on a cluster epidemic of COVID-19 in a supermarket in Liaocheng, Shandong province. Chin J Epidemiol. (2020) 41:2024–8. 10.3760/cma.j.cn112338-20200228-00206 32340093

[11] Zhao Han LB Xia Yu. Zhou Hailong, Li Tingrong, Zeng Yi, Zhu Xiaoling, Zhou Yuxiang, Li Qin. Investigation of transmission chain of a cluster COVID-19 cases. Chin J Epidemiol. (2020) 41:2015–9. 10.3760/cma.j.cn112338-20200227-00198 32397697

[12] Leclerc QJ Fuller NM Knight LE Funk S Knight GM. What settings have been linked to SARS-CoV-2 transmission clusters? Wellcome Open Res. (2020) 5:83. 10.12688/wellcomeopenres.15889.2 32656368 PMC7327724

[13] Pan Xingqiang CY Wang A Wang J Ye L Gu S Fang T. Study on transmission dynamic of 15 clusters of COVID-2019 cases in Ningbo. Chin J Epidemiol. (2020) 41:2010–4. 10.3760/cma.j.cn112338-20200330-00466 32397699

[14] Jing Qinlong LY Ma M Gu Y Li K Ma Y Wu D. Contagiousness and secondary attack rate of 2019 novel coronavirus based on cluster epidemics of COVID-19 in Guangzhou. Chin J Epidemiol. (2020) 41:1623–6. 32388933 10.3760/cma.j.cn112338-20200310-00305

[15] Meyerowitz EA Richterman A Gandhi RT Sax PE. Transmission of SARS-CoV-2: a review of viral, host, and environmental factors. Ann Intern Med. (2021) 174:69–79. 10.7326/M20-5008 32941052 PMC7505025

[16] Lessler, J, Grantz, K,. Overdispersion of COVID-19. Johns Hopkins Bloomberg School of Public Health. Available online at: https://www.jhsph.edu/covid-19/articles/overdispersion-of-covid-19.html (accessed May 11, 2021).

[17] Liu T Gong D Xiao J Hu J He G Rong Z. Cluster infections play important roles in the rapid evolution of COVID-19 transmission: a systematic review. Int J Infect Dis. (2020) 99:374–80. 10.1016/j.ijid.2020.07.073 32768702 PMC7405860

[18] Chen T Zhao Z Rui J Yu S Zhu Y Xu J. Estimating the transmissibility of coronavirus disease 2019 and assessing the effectiveness of the countermeasures to control the disease in Xiamen City. Journal of Xiamen University. (2020) 59:298–303. 10.6043/j.issn.0438-0479.202003003

[19] Zhao ZY Zhu YZ Xu JW Hu SX Hu QQ Lei Z. A five-compartment model of age-specific transmissibility of SARS-CoV-2. Infect Dis Poverty. (2020) 9:117. 10.1186/s40249-020-00735-x 32843094 PMC7447599

[20] Zhao Q Yang M Wang Y Yao L Chen T. Effectiveness of Interventions to Control Transmission of Reemergent Cases of COVID-19—Jilin Province, China, 2020. China CDC Weekly. (2020) 2:651–4. 10.46234/ccdcw2020.181 34594730 PMC8422243

[21] Dietz K. The estimation of the basic reproduction number for infectious diseases. Stat Methods Med Res. (1993) 2:23–41. 10.1177/096228029300200103 8261248

[22] DCEPH National notifiable infectious disease database [in Chinese] (2018). Available online at: http://www.phsciencedata.cn/Share/ky_sjml.jsp (accessed December, 2019).

[23] General General Office of National Health Commission Office Office of National Administration of Traditional Chinese Medicine. Protocol of diagnosis and treatment for COVID-19(trial version 8). China Med. (2020) 15:1494–9. 10.1097/01.ID9.0000733564.2178

[24] Chen TM Rui J Wang QP Zhao ZY Cui JA Yin L. A mathematical model for simulating the phase-based transmissibility of a novel coronavirus. Infect Dis Poverty. (2020) 9:24. 10.1186/s40249-020-00640-3 32111262 PMC7047374

[25] Zhang S Hu Q Deng Z Hu S Liu F Yu S. Transmissibility of acute haemorrhagic conjunctivitis in small-scale outbreaks in Hunan Province, China. Sci Rep. (2020) 10:119. 10.1038/s41598-019-56850-9 31924848 PMC6954223

[26] Chunna M Shuangsheng W Ying S Yi Z Wei D Wenting W. Characteristics of COVID-19 epidemics at different stages in Beijing from January to July, 2020. Int J Virol. (2020) 27:448–54. 10.3760/cma.j.issn.1673-4092.2020.06.003

[27] Lauer SA Bi GK QF. The incubation period of 2019-nCoV from publicly reported confirmed cases: estimation and application. medRxiv [Preprint]. (2020). 10.1101/2020.02.02.20020016 32150748 PMC7081172

[28] Linton NM Kobayashi T Yang Y Hayashi K Akhmetzhanov AR Jung SM. Incubation Period and Other Epidemiological Characteristics of 2019 Novel Coronavirus Infections with Right Truncation: A Statistical Analysis of Publicly Available Case Data. J Clin Med. (2020) 9. 10.3390/jcm9020538 32079150 PMC7074197

[29] Backer JA Klinkenberg D Wallinga J. Incubation period of 2019 novel coronavirus (2019-nCoV) infections among travellers from Wuhan, China, 20-28 January 2020. Euro Surveill. (2020) 25. 10.2807/1560-7917.ES.2020.25.5.2000062 32046819 PMC7014672

[30] Wang P LJ Jin YY. Epidemiological characteristics of 1212 COVID-19 patients in Henan, China. medRxiv [Preprint]. (2020). 10.1101/2020.02.21.20026112

[31] Chan JF Yuan S Kok KH To KK Chu H Yang J. A familial cluster of pneumonia associated with the 2019 novel coronavirus indicating person-to-person transmission: a study of a family cluster. Lancet. (2020) 395:514–23. 10.1016/S0140-6736(20)30154-9 31986261 PMC7159286

[32] Liu YF LJ Zhou PH. Analysis on cluster cases of COVID-19 in Tianjin. Chin J Epidemiol. (2020) 41:654–7. 10.3760/cma.j.cn112338-20200225-00165 32213269

[33] Park SY Kim YM Yi S Lee S Na BJ Kim CB. Coronavirus disease outbreak in call center, South Korea. Emerg Infect Dis. (2020) 26:1666–70. 10.3201/eid2608.201274 32324530 PMC7392450

[34] Dyal JW Grant MP Broadwater K Bjork A Waltenburg MA Gibbins JD. COVID-19 Among Workers in Meat and Poultry Processing Facilities-19 States, April 2020. MMWR. (2020) 69. 10.15585/mmwr.mm6918e3 32379731

[35] U.S. Centers for Disease Control and Prevention, Occupational Safety and Health Administration. Meat and Poultry Processing Workers and Employers. Atlanta: CDC (2020). Available online at: https://www.cdc.gov/coronavirus/2019-ncov/community/organizations/meat-poultryprocessing-workers-employers.html.

[36] Zhang Wenting LD Xie C Shen D Chen Z Li Z Liu Y. Sensitivity and specificity of nucleic acid testing in close contacts of COVID-19 cases in Guangzhou. Chin J Epidemiol. (2021) 42:1347–52. 10.3760/cma.j.cn112338-20201211-01400 34814552

[37] Yang Haiyan XJ Li Y Liang X Jin Y Chen S Zhang R. The preliminary analysis on the characteristics of the cluster for the COVID-19 Chin J Epidemiol. (2020) 41:623–8. 10.3760/cma.j.cn112338-20200223-00153 32145716

[38] Hao X Cheng S Wu D Wu T Lin X Wang C. Reconstruction of the full transmission dynamics of COVID-19 in Wuhan. Nature. (2020) 584:420–4. 10.1038/s41586-020-2554-8 32674112

[39] Su L Hong N Zhou X He J Ma Y Jiang H. Evaluation of the secondary transmission pattern and epidemic prediction of COVID-19 in the four metropolitan areas of China. Front Med. (2020) 7:171. 10.3389/fmed.2020.00171 32574319 PMC7221060

[40] Leung K Wu JT Liu D Leung GM. First-wave COVID-19 transmissibility and severity in China outside Hubei after control measures, and second-wave scenario planning: a modelling impact assessment. Lancet. (2020) 395:1382–93. 10.1016/S0140-6736(20)30746-7 32277878 PMC7195331

[41] Gumel AB Ruan S Day T Watmough J Brauer F van den Driessche P. Modelling strategies for controlling SARS outbreaks. Proc Biol Sci. (2004) 271:2223–32. 10.1098/rspb.2004.2800 15539347 PMC1691853

[42] Majumder MS Rivers C Lofgren E Fisman D. Estimation of MERS-coronavirus reproductive number and case fatality rate for the spring 2014 Saudi Arabia outbreak: insights from publicly available data. PLoS Curr. (2014) 6. 10.1371/currents.outbreaks.98d2f8f3382d84f390736cd5f5fe133c 25685622 PMC4322060

[43] Chen TM Chen QP Liu RC Szot A Chen SL Zhao J. The transmissibility estimation of influenza with early stage data of small-scale outbreaks in Changsha, China, 2005–2013. Epidemiol Infect. (2017) 145:424–33. 10.1017/S0950268816002508 27834157 PMC5244440

[44] Waltenburg MA Victoroff T Rose CE Butterfield M Jervis RH Fedak KM. Update: COVID-19 among workers in meat and poultry processing facilities-United States, April-May 2020. MMWR. (2020) 69:887–92. 32644986 10.15585/mmwr.mm6927e2 PMC7732361

[45] Schweizer C Edwards RD Bayer-Oglesby L Gauderman WJ Ilacqua V Jantunen MJ. Indoor time-microenvironment-activity patterns in seven regions of Europe. J Expo Sci Environ Epidemiol. (2007) 17:170–81. 10.1038/sj.jes.7500490 16721413

[46] Rothe C Schunk M Sothmann P Bretzel G Froeschl G Wallrauch C. Transmission of 2019-nCoV infection from an asymptomatic contact in Germany. N Engl J Med. (2020) 382:970–1. 10.1056/NEJMc2001468 32003551 PMC7120970

[47] Guan J Wei Y Zhao Y Chen F. Modeling the transmission dynamics of COVID-19 epidemic: a systematic review. J Biomed Res. (2020) 34:422–30. 10.7555/JBR.34.20200119 33243940 PMC7718076

[48] Wikipedia:Basic reproduction number. Available online at: https://en.wikipedia.org/wiki/Basic_reproduction_number#Effective_reproduction_number (accessed October 2, 2021).

[49] Shen Litong DZ Chen Z Yang T Lin T Zhang R Jiang L. Effectiveness of the “14 plus 7 day quarantine” and “nucleic acid plus total antibody testing” strategy for screening imported patients with COVID-19 in Xiamen. Chin J Epidemiol. (2021) 42:1002–7. 10.3760/cma.j.cn112338-20210128-00076 34814497

[50] Lou B Li TD Zheng SF Su YY Li ZY Liu W. Serology characteristics of SARS-CoV-2 infection after exposure and post-symptom onset. Eur Respir J. (2020) 56. 10.1183/13993003.00763-2020 32430429 PMC7401320

[51] Deeks JJ Dinnes J Takwoingi Y Davenport C Spijker R Taylor-Phillips S. Antibody tests for identification of current and past infection with SARS-CoV-2. Cochrane Database Syst Rev. (2020) 6:Cd013652. 10.1002/14651858.CD013652 32584464 PMC7387103

[52] Wei YY Lu Z Du ZC Zhang ZJ Zhao Y Shen SP. Fitting and forecasting the trend of COVID-19 by SEIR+CAQdynamic model. Chin J Epidemiol. (2020) 41:470–5. 10.3760/cma.j.cn112338-20200216-00106 32113198

[53] Liu KQ YY Liu ZX. The impact of meteorological and environmental conditions on the spread of COVID-19. J Pub Health Prev Med. (2020) 31:9–13 10.3969/i.issn.1006-2483.2020.04.003

[54] Pathak EB Salemi JL Sobers N Menard J Hambleton IR. COVID-19 in children in the United States: intensive care admissions, estimated total infected, and projected numbers of severe pediatric cases in 2020. J Public Health Manag Pract. (2020) 26:325–33. 10.1097/PHH.0000000000001190 32282440 PMC7172976

