# Supplementary material for: Epidemiological Characteristics and Transmissibility for SARS-CoV-2 of Population Level and Cluster Level in a Chinese City
Source: Front Public Health. 2022 Jan 18;9:799536. doi: 10.3389/fpubh.2021.799536 (PMC8805998; doi:10.3389/fpubh.2021.799536)
Supplement: Supplementary file 1 [file Data_Sheet_1.docx]

Supplementary Material

**Model selection**

The most commonly used models in COVID-19 are susceptible-infectious-removed model (SIR), susceptible-exposed-infectious-removed model (SEIR)(1). The classic SIR model includes 3 compartments: susceptible (S), infectious (I), removed (R). However, infectivity can occur 1-3 days before the onset of symptoms. Infected persons can spread the disease even if they are pre-symptomatic or asymptomatic(2). And a certain proportion of asymptomatic infections have been found(3). Our study added an exposed (E) and asymptomatic (A) compartment on the basis of SEIR model, and we established the susceptible-exposed-infectious-asymptomatic-removed model (SEIAR)

**Facts and Assumptions of SEIAR model**

1. Natural birth rate and death rate will lead to negligible changes for the population in a short time. Thus, we didn’t consider such birth rate and natural death rate.
2. Assuming that the infection rate coefficient after effective contact between *S* and *I* is *β*, and assuming that *A* is infectious, and the transmissibility is *κ* (0<*κ*<1) times that of *I*, then at time *t*, the number of new infections is *βS*(*I+κA*).
3. Assuming that the proportion of asymptomatic infection is *p*, and the incubation period (period between exposure and onset of clinical symptoms) and latent period (period between exposure and ability to transmit to others) are 1/*ω* and 1/*ω'*, respectively. At time *t*, the number of people who change from *E* to *A* and I are *pω'E* and (1-*p*) *ωE*, respectively.
4. Assuming that the infectious period (the length of time for which an infected individual is infectious to others) of the case *I* to the first diagnosis is 1/*γ*, then at time *t*, the number of people who change from *I* to *R* is *γI*. Because there are death cases in the reported data, we set *f* as the case fatality rate, then the number of deaths at time t is *fI*.
5. Assuming that the infectious period of the recessive infected person *A* is 1/*γ'*, then at time *t*, the number of people who change from *A* to *R* is *γ'A*.
6. Once a patient is infected and cured, there will be no secondary infections.

Based on these assumptions, the equations of the SEIAR model are presented as follows:

$$\frac{dS}{dt}=-\beta S\left( I+kA \right)$$

$$\frac{dE}{dt}=\beta S\left( I+kA \right)-p\omega^{'}E-\left( 1-p \right)\omega E$$

$$\frac{dI}{dt}=np+\left( 1-p \right)\omega E-\gamma I-fI$$

$$\frac{dA}{dt}=p\omega^{'}E-\gamma^{'}A$$

$$\frac{dR}{dt}=\gamma I+\gamma'A$$

$$x=\left( 1-p \right)\omega E$$

$N=S+E+I+A+R$ （1）

In the model, the total population (*N*) was divided into five parts: susceptible (*S*), exposed (*E*), infectious (*I*), asymptomatic (*A*) and removed (*R*), and *dS/dt*、*dE/dt*、*dI/dt*、*dA/dt*、*dR/dt* represent the change rate of *S*, *E*, *I*, *A* and *R* at *t* time, respectively.

**Parameter estimation for SEIAR model**

The infection rate coefficient *β* was estimated by fitting the model with actual data. Actual data calculation results were used when estimating parameters such as *p* (asymptomatic infection proportion), *f* (case fatality rate), incubation period (1/*ω*), latent period (1/*ω’*), infectious period of symptomatic infection (1/*γ*) and infectious period of asymptomatic persons (1/*γ* '). We assumed that the transmissibility of asymptomatic infection was 0.5 times that of symptomatic infection (к = 0.5) from information in a previous study(4).

**Inclusion and exclusion criteria of clusters**

In our study, clusters were collected that met the following inclusion and exclusion criteria.

1. Clusters: there were 2 or more cases of fever and/or respiratory symptoms in small areas such as homes, offices, school classes within 2 weeks(5).”
2. At least one secondary case is a local case or an import-related case.
3. The source of infection should be identified for the primary case, whether it is a local case or an imported case. If the primary case is a case with an unknown source of infection, for example, if it is an infection gathering in places where the chain of transmission cannot be traced, it will be excluded.

The case definition according to the source of infection is as follows.

1. Imported case: There is clear evidence that the infection occurred outside of Beijing City.
2. Local case: There is a clear history of contact with confirmed cases in Beijing, or it can be proved that the infection occurred in Beijing.
3. Case of unknown source of infection: After inquiry and investigation, no clear source of infection and exposure history were found.

According to the inclusion and exclusion criteria established in this article (Supplementary Figure S1), 10 cases in 4 clusters were excluded because there were no secondary cases in these clusters. Subsequently, one cluster of 71 cases was excluded from 24 clusters for unknown sources of infection. The reason was that there were 2 or more imported cases in each cluster and it is impossible to clarify the primary case. Eventually, 9 clusters were finally analyzed, involving 31 secondary cases and 9 first cases.

**Definition of *R*_0_**

*R*_0_ is defined as“the average number of secondary infections produced when one infected individual is introduced into a host population where everyone is susceptible”, as phrased by Anderson and May(6). *R*_0_ was calculated by using individual-level contact tracing data obtained at the onset of the epidemic(7). Once an individual is diagnosed, his/her contacts are traced and tested. *R*_0_ is then computed by averaging over the number of secondary cases of many diagnosed individuals.

**References**

1. Guan J, Wei Y, Zhao Y, Chen F. Modeling the transmission dynamics of COVID-19 epidemic: a systematic review. *J Biomed Res* (2020) 34(6):422-30. Epub 2020/11/28. doi: 10.7555/jbr.34.20200119. PubMed PMID: 33243940; PubMed Central PMCID: PMCPMC7718076.

2. Communicable Diseases Network Australia. "Coronavirus Disease 2019 (COVID-19): CDNA National Guidelines for Public Health Units". 5.1. Communicable Diseases Network Australia/Australian Government Department of Health. <https://www1.health.gov.au/internet/main/publishing.nsf/Content/cdna-song-novel-coronavirus.htm> accessed by December 1,2021.

3. Li R, Pei S, Chen B, Song Y, Zhang T, Yang W, et al. Substantial undocumented infection facilitates the rapid dissemination of novel coronavirus (SARS-CoV-2). *Science* (2020) 368(6490):489-93. Epub 2020/03/18. doi: 10.1126/science.abb3221. PubMed PMID: 32179701; PubMed Central PMCID: PMCPMC7164387.

4. Chen TM, Rui J, Wang QP, Zhao ZY, Cui JA, Yin L. A mathematical model for simulating the phase-based transmissibility of a novel coronavirus. *Infect Dis Poverty* (2020) 9(1):24. Epub 2020/03/01. doi: 10.1186/s40249-020-00640-3. PubMed PMID: 32111262; PubMed Central PMCID: PMCPMC7047374.

5. General Office of National Health Commission, Office of National Administration of Traditional Chinese Medicine. Protocol of diagnosis and treatment for COVID-19(trial version 8). *China Med* (2020) 15(10):1494-9. doi: 10.3760/j.issn.1673-4777.2020.10.002.

6. Anderson R MR. *Infectious Diseases of Humans.* . Oxford: Oxford University Press (1992).

7. Breban R, Vardavas R, Blower S. Theory versus data: how to calculate R0? *PLoS One* (2007) 2(3):e282. Epub 2007/03/16. doi: 10.1371/journal.pone.0000282. PubMed PMID: 17356693; PubMed Central PMCID: PMCPMC1804098.

**Supplementary Figure S1. Flowchart for showing the inclusion and exclusion of clusters**


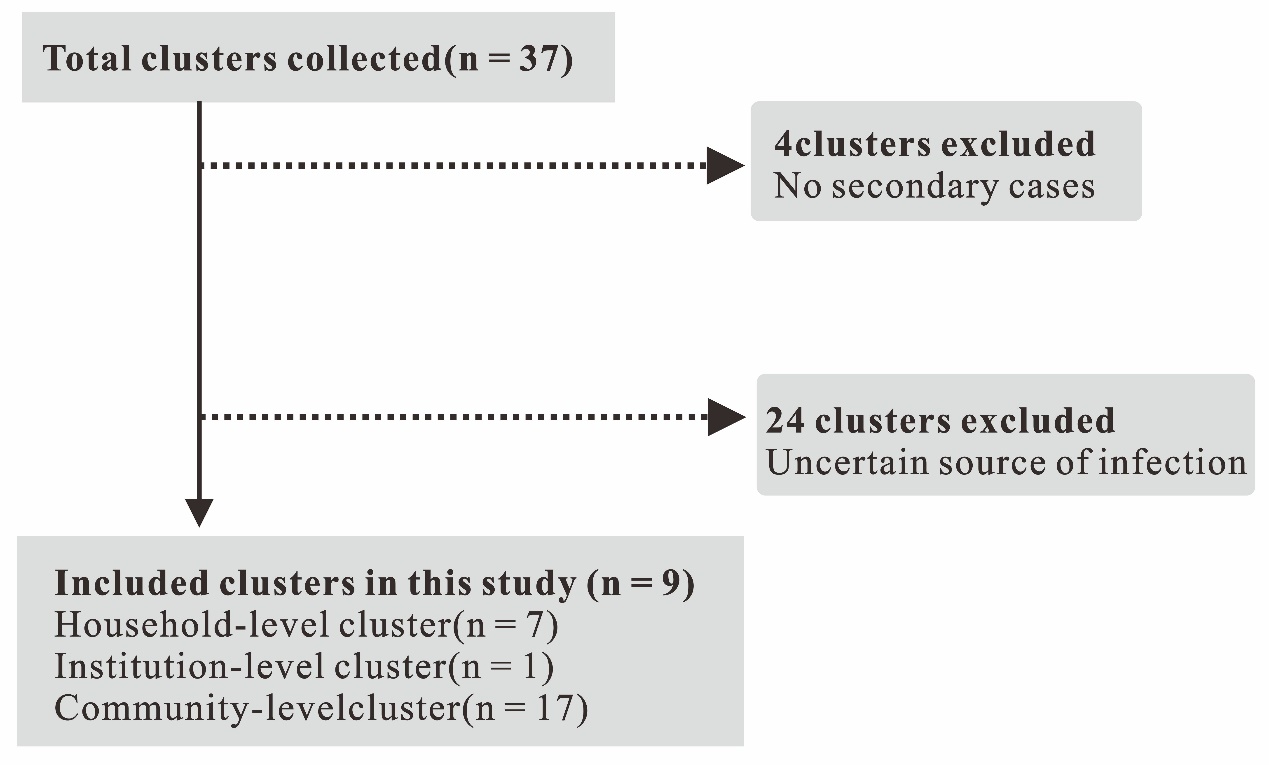


**Supplementary Figure S2. Schematic diagram for showing the calculation of *R*_0_ based on definition**


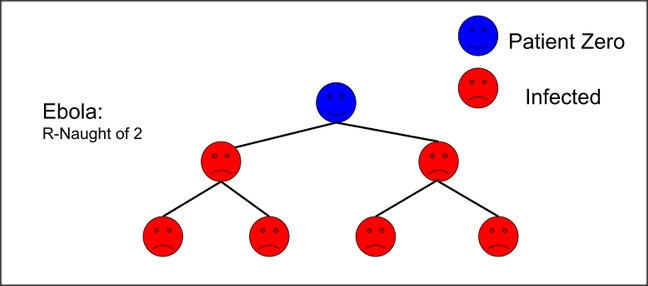


This file is from the Wikimedia Commons (<https://en.wikipedia.org/wiki/File:R_Naught_Ebola_and_Flu_Diagram.svg>).
